# Collaboration amongst general practitioners and gynaecologists working in primary health care in Germany: a cross-sectional study

**DOI:** 10.1017/S1463423621000165

**Published:** 2020-05-06

**Authors:** Barbara Trusch, Christoph Heintze, Elena Petelos, Lorena Dini

**Affiliations:** 1 Charité – Universitätsmedizin Berlin, Corporate Member of Freie Universität Berlin, Humboldt-Universität zu Berlin, and Berlin Institute of Health, Institute of General Practice, Charitéplatz 1, Berlin, Germany; 2 Clinic of Social and Family Medicine, Department of Social Medicine, Faculty of Medicine, University of Crete, Heraklion, Crete, Greece

**Keywords:** collaboration, general practitioners, gynaecology, outpatient care, primary health care, women’s health

## Abstract

**Aim:**

This cross-sectional study is the first one to explore the collaboration of the influencing factors thereof amongst general practitioners (GPs) and gynaecologists (Gyns) working in primary care in urban and rural settings in Germany.

**Background:**

The number of women aged ≥ 50 years is predicted to increase in the next years in Germany. This coincides with the ageing of primary care specialists providing outpatient care. Whereas delegation of tasks to nurses as a form of interprofessional collaboration has been the target of recent studies, there is no data regarding collaboration amongst physicians in different specialisations working in primary care. We explored collaboration amongst GPs and Gyn regarding the healthcare provision to women aged ≥ 50 years.

**Methods:**

A quantitative postal survey was administered to GPs and Gyns in three federal states in Germany, focusing on care provision to women aged ≥ 50 years. A total of 4545 physicians, comprising 3514 GPs (67% of the total GP population) randomly selected, and all 1031 Gyns practicing in these states received the postal survey in March 2018. A single reminder was sent in April 2018 with data collection ending in June 2018. Multiple logistic regressions were performed for collaboration, adjusted by age and sex, alongside descriptive methods.

**Findings:**

The overall response rate was 31% (1389 respondents): 861 GPs (25%) and 528 Gyns (51%), with the mean respondent age being 54.4 years. Seventy-two per cent were female. Key competencies of collaboration are associated with working in rural federal states and with network participation. Physicians from rural states [odds ratio (OR) = 1.5, 95% confidence interval (CI) = 1.2, 1.9] and physicians in networks (OR = 3.0, CI = 2.3, 3.9) were more satisfied with collaboration. Collaboration to deliver services for women aged ≥ 50 years is more systematic amongst GPs and Gyns who are members of a network; increased networking could improve collaboration, and ultimately, outcomes too.

## Introduction

In Germany, utilisation of healthcare services varies with patient age (Robert Koch-Institute, 2014; Krause & Prütz, 2020). Annual visits to gynaecologists (Gyns) are more common at a younger age, whereas 75% of women between 40 and 49 years continue to report annual visits. However, after the age of 50, the number of women visiting their Gyn annually drops, with a steady decrease to below 45% in the group of 70–79 years. Interestingly, it is not an effect reflected in the utilisation of general practitioner (GP) services amongst women aged ≥ 50 years, with the visiting rate remaining stable at 80% across all age groups for women aged ≥ 50 years (Rattay *et al.*, 2013; Krause & Prütz, 2020).

From a public health perspective, the health needs of women aged ≥ 50 years deserve to be identified and addressed by the healthcare system through the provision of gynaecological care services for women of all ages (Tannenbaum *et al.*, 2003). Indeed, the Sustainable Development Goals (SDGs) of the United Nations make an explicit reference to age in SDG 3: ensure healthy lives and promote well-being for all at all ages, whereas the framework provided aims to address gender equality and improvement of health and well-being for all women throughout the life course, with the strategy and action plans for Europe showing that even in developed countries, a lot of progress needs to be made to meet the goals (World Health Organization Regional Office for Europe, 2016).

## Background

In Germany, outpatient services can only be provided by specialised physicians including GPs, and Gyns providing outpatient care. GPs and Gyns can either be self-employed practice owners, holding a seat in the Regional Association of Statutory Health Insurance (SHI), or work as employees (Advisory Council on the Assessment of Developments in the Healthcare Sector, 2018). Service provision is regulated on the basis of speciality including remuneration for SHI physicians. GPs cannot charge for gynaecological services, nor provide services not included in their predetermined service catalogue (National Association of Statutory Health Insurance Physicians, 2005; German Medical Association, 2020). In consequence, women have to visit a Gyn, for example, for gynaecological cancer screening or sexual health issues. Women can either go straight to consult a Gyn or visit a GP first, who may act as a gatekeeper deciding on further referrals.

Over the past decade, GP shortages have increased across Europe, including in the United Kingdom (Iacobucci, 2019), in Denmark (Kirstine Andersen *et al.*, 2018), in France and Switzerland (Cerny *et al.*, 2016), and in Hungary (Papp *et al.*, 2019). Especially in rural areas, age-related retirement of GPs coincides with a restrained interest of younger doctors to become SHI physicians and establish their own practice (Robinson *et al.*, 2010; Dini *et al.*, 2012; Iacobucci, 2014; Kjosavik, 2018; Advisory Council on the Assessment of Developments in the Healthcare Sector, 2018). In the North-Eastern region of Germany, which includes the federal state of Berlin, an urban area with a population density of 4090 inhabitants per m^2^, as well as the rural federal states of Brandenburg and Mecklenburg-Western Pomerania which have the lowest population density in Germany (85, respectively, 69 inhabitants per m²), one-third of the GPs and the Gyns working in outpatient care are aged ≥ 60 years (German Medical Association, 2017; Federal Statistical Office of Germany, 2020). With young doctors tending to move to urban areas, many medical practices in rural areas remain vacant across regions (Advisory Council on the Assessment of Developments in the Healthcare Sector, 2018).

**Figure 1.**
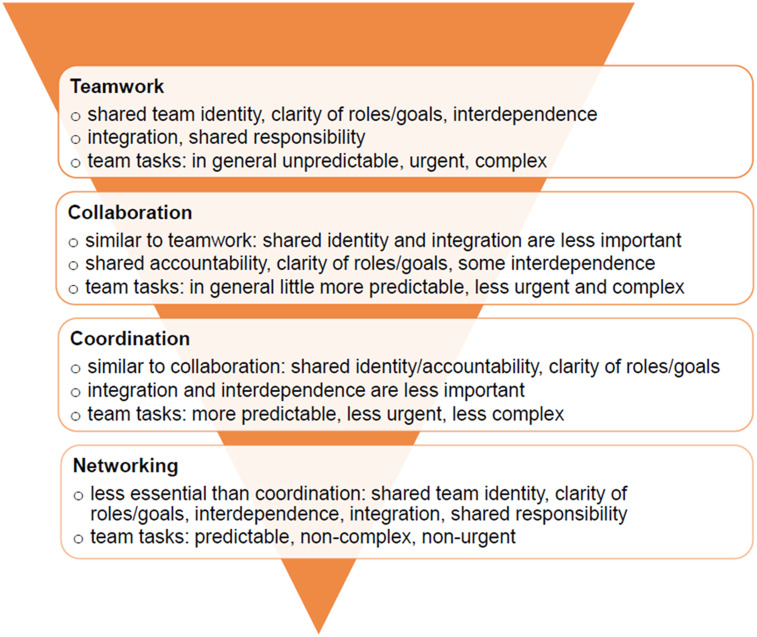
Interprofessional practices: the contingency approach, modified from Reeves *et al.*, 2018

To address shortages, old concepts based on delegation, substitution, and telemedicine have been rediscovered and explored internationally, that is, in the context of general practice, or nursing (Wijers *et al.*, 2012; Friman *et al.*, 2018; Dini *et al.*, 2020; Döpfmer *et al.*, 2020). However, collaboration and the way it shapes practice has been explored less systematically. Reeves *et al.* (2018) defined interprofessional practice as a ‘contingency approach’ based on four categories in the continuum of care: teamwork, collaboration, coordination, and networking (see Figure 1). The position in this continuum is determined by a shared team identity, clear roles, and goals, interdependence, integration, shared responsibility, and through the predictability, urgency, and complexity of team tasks, where teamwork, collaboration, coordination, and networking are nested in each other (Xyrichis *et al.*, 2018). Previous studies have addressed three of the four categories of interprofessional practices: The role of teamwork has been explored in relation to the concept of delegation and in the context of teams and the degree of their patient-centredness as well as communication and co-treatment (Dini *et al.*, 2012; Bennett *et al.*, 2015; Donnelly *et al.*, 2019). Coordination is increasingly playing a role to overcome sectoral boundaries in outpatient care (Burkhardt & Trojan, 2018; Gödde *et al.*, 2018).

So far, little is known about collaboration as a core competency of primary healthcare specialists (Beaulieu *et al.*, 2009). The Medical Competencies Framework of the Royal College of Physicians and Surgeons of Canada (CanMEDs) defines collaboration as ‘effective work with other care professionals to provide safe, high-quality, and patient-centred care’. Three key competencies are described for the collaborator: ‘Work effectively with physicians and other colleagues in the healthcare professions (Work effectively with physicians)’, ‘Hand over the care of a patient to another healthcare professional to facilitate continuity of safe patient care (Continuity of safe patient care)’, and ‘Work with physicians and other colleagues in the healthcare professions to promote understanding, manage differences, and resolve conflicts (Promote understanding)’ (Richardson *et al.*, 2015).

The aim of this cross-sectional study is to explore the key competencies of collaboration and its influencing factors for GPs and Gyns, both working as specialists in primary care in urban and rural settings.

## Methods

We conducted a quantitative cross-sectional study by administering an anonymous postal survey for GPs and Gyns practising in three key federal states of the North-Eastern region of Germany.

### Survey population, sampling frame, and data collection

We obtained contact details of GPs and Gyns from the official registry (November 2017–February 2018) of the regional SHI associations of each of the three federal states.

After quality control of the obtained directories, the total number of GPs included was 5265, whereas the total number of Gyns in primary care providing outpatient services in the region was 1031, excluding those providing only fertility services. To ensure representative responses for both speciality groups, and considering their geographical distribution, we chose two different survey strategies. Based on previous cooperation rates for the purposes of completing GP surveys in Germany, and to obtain a response of at least 10% of the total population of the GPs in the three federal states, we calculated the sample size needed, surveying a randomly selected sample of 67% of all GPs and conducting a comprehensive survey including all Gyns. We contacted a total of 4545 physicians. A list of random numbers was generated with the R software using a seed value and proceeding with the random allocation of numbers to the alphabetically sorted address list. Random sampling of GPs was stratified by state, district, and sex. Within each stratum, the first 67% of GPs were drawn in descending order of randomly allocated numbers.

The survey followed the total method design with one reminder (de Leeuw & Hox, 2008; Dini *et al.*, 2020). We conducted a monitoring of responses using non-anonymous response postcards that were already included in the survey envelope alongside the explanatory cover letter and the questionnaire. Physicians who had not sent their response postcard by mid-April 2018 received the reminder letter and survey documents again. Participation was voluntary and no incentives or any form of compensation was provided for participants. The data collection was conducted from March to June 2018.

### Survey instrument

We developed a one-sheet double-sided paper-and-pencil questionnaire, for both GPs and Gyns, based on previous surveys in Germany, addressing delegation amongst GPs (Dini *et al.*, 2012; Richardson *et al.*, 2015; Dini *et al*., 2020; Reeves *et al.*, 2018). We piloted the questionnaire with a small group of 20 Gyns and GPs, as well as with representatives of regional professional associations. The final questionnaire was created with the Teleform software (Electric Paper Informationssysteme GmbH, 2007) as a scannable form, and the pilot demonstrated could be completed in under 20 min.

Data collection included personal data (e.g., age, sex, employment status), practice of networking (being member of a network), medical office data (e.g., type of practice, state), and work environment data (e.g., working hours, perceived workload).

**Figure 2.**
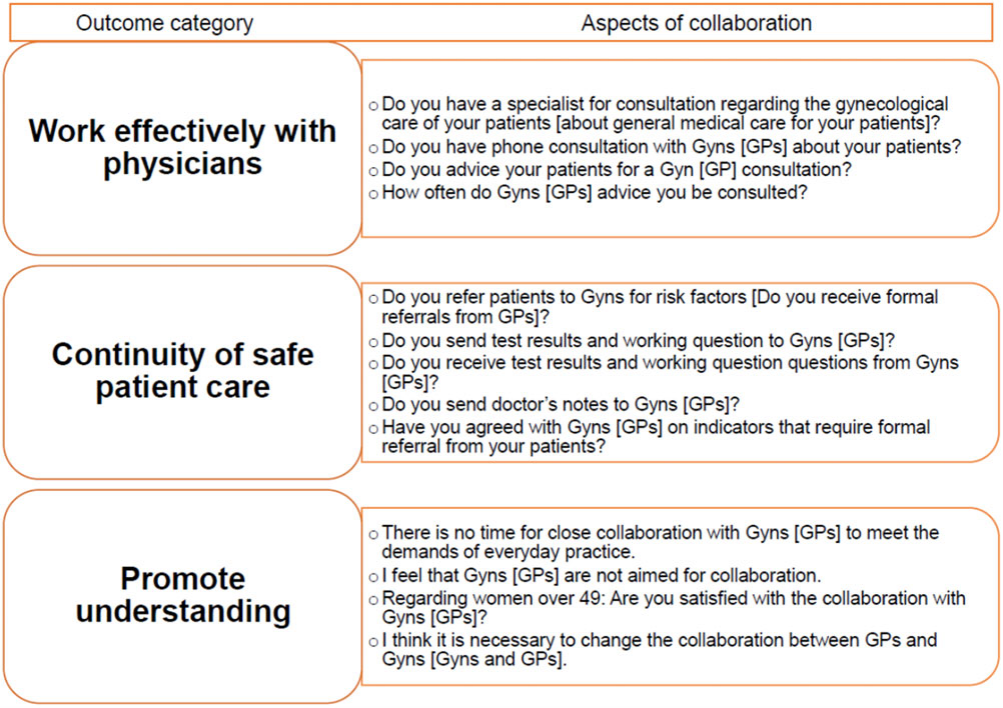
Classification of aspects of collaboration assigned to the key competencies of CanMEDs. Questions for GPs are shown, wording for Gyns bracketed

The main outcomes of this study concern the competencies of collaboration assessed with 13 aspects according to the 3 CanMEDs key competencies of collaborator: for ‘Work effectively with physicians’ and ‘Continuity of safe patient care’, we used a five-level scale (always, often, rarely, not at all, do not need) and for ‘Promote understanding’, a six-level scale (yes, mostly yes, partly, mostly no, no, do not know). The outcome ‘Work effectively with physicians’ included questions on current collaboration, ‘Continuity of safe patient care’ on patient or patient data transfer between specialists, and ‘Promote understanding’ on satisfaction with collaboration, barriers, and considering a change in collaboration needed (see Figure 2).

### Data analysis

Questionnaires received were scanned and verified with the software Teleform (Electric Paper Informationssysteme GmbH, 2007) and imported into a database for analysis with IBM Statistics SPSS 25. Data control and plausibility checks were independently carried out by two researchers. The database was cross-checked against the paper questionnaire, and manually corrected in case of identified discrepancies. The proportion of missing values corresponded to less than 6% of the unanswered questions; these were classified as missing completely at random. Therefore, we used a list-wise deletion for the analysis (Rässler *et al.*, 2008).

Collaboration is reported for 13 aspects organised along with the 3 categories of collaboration as comparison of ‘frequently (always/frequently) versus rarely (rarely/not at all/do not need)’ for ‘work effectively with physicians (4 aspects)’ and ‘continuity of safe patient care (5 aspects)’, and as ‘mostly yes (yes/mostly yes) versus mostly no (partly/mostly no/no)’ without consideration of “do not know” for “promote understanding (4 aspects, see Figure 2)”. We classified the three federal states on the basis on their characteristics as ‘urban’ (Berlin) and ‘rural’ (Brandenburg and Mecklenburg-Western Pomerania). Being member of a network with the other group of specialists was assumed to be true if at least one of the three items for formal networking (e.g., in a quality circle) or the two items for informal networking (e.g., through personal contact) was selected. This was contrasted with the item ‘not at all’ for the lack of a network. Type of practices are reported as ‘solo practice’ versus ‘other’ (joint practices or medical care centres), and a perceived high workload as high (very high/high) or low (appropriate/rather low/low), excluding the answer ‘do not know’.

We used descriptive methods, multiple logistic regressions to characterise groups and their relations, and two-sided chi-square tests to explore differences. Additionally, we compared the mean values of characteristics for both groups of respondents to those of the regional physician population for both groups to assess external validity and response bias.

We computed multiple logistic regressions for all 13 aspects of the key competencies of collaboration and calculated odds ratios (ORs). The probability of error was set at 5%. Each regression model contained one of these aspects as the dependent variable adjusted for age and sex. Additional covariates for the multiple logistic regressions were rural/urban characteristics, reported average weekly working hours, perceived workload, type of practice, and the networking practice. Models were calculated and fitted according to best predicting variables. We reported OR in all models with their confidence intervals (CI) at 95%. The results of the descriptive methods are reported unweighted (number and percentages) and the results of regressions weighted (percentages).

## Results

**Figure 3.**
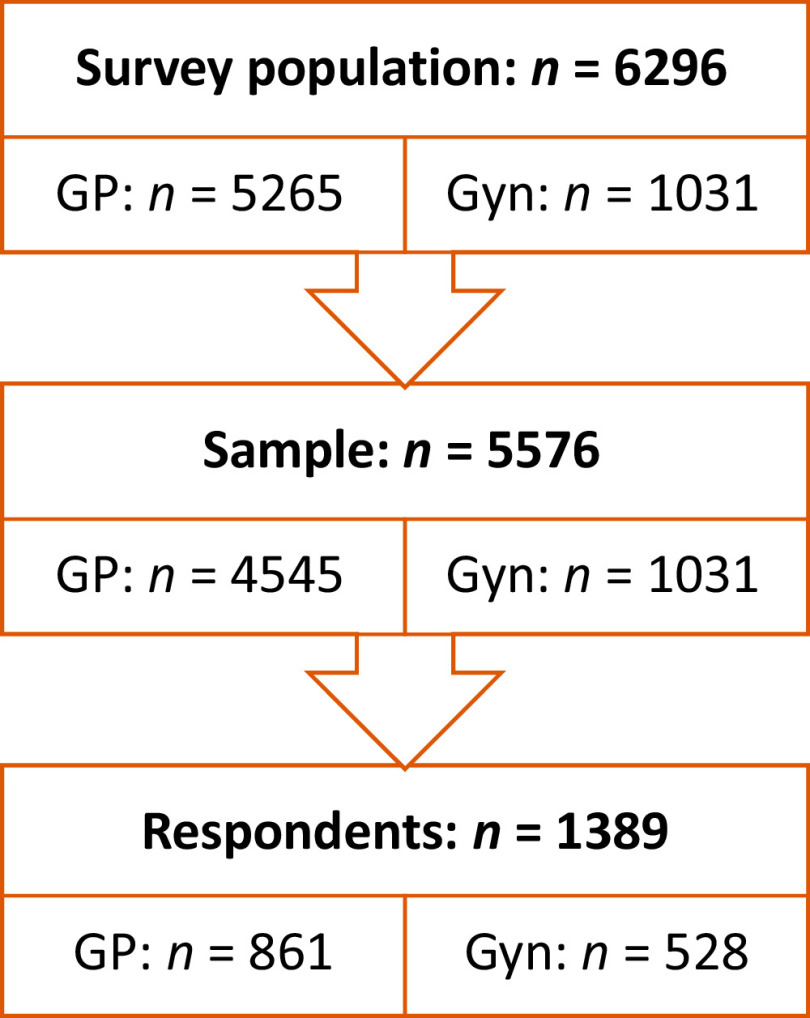
Population, sample, and respondents. GP = General practitioners; Gyn = Gynaecologists

From the contacted physicians, 1389 agreed to participate, completed, and returned the questionnaire (see Figure 3). The overall response rate achieved was 31%. Speciality-specific response rates were 25% (*n* = 861) for GPs and 51% (*n* = 528) for contacted Gyns. As shown in Table 1, most respondents were working in rural areas (55%), with the majority being female (72%) and practice owners (80%).

**Table 1.** Characteristics of respondents

Characteristics of respondents	All respondents (*n* = 1389)		General practitioners (*n* = 861)		Gynaecologists (*n* = 528)	
Age in years (Mean, SD)	54.4	(8.9)	54.4	(9.2)	54.3	(8.3)
Sex						
Male (*n*, %)	374	(28.4)	270	(33.1)	104	(20.8)
Female (*n*, %)	942	(71.6)	546	(66.9)	396	(79.2)
Type of federal state						
Urban (*n*, %)	602	(45.0)	367	(44.1)	235	(46.4)
Rural (*n*, %)	737	(55.0)	466	(55.9)	271	(53.6)
Networking						
Member of a network (*n*, %)	941	(67,7)	540	(62.7)	401	(75.9)
No network (*n*, %)	448	(32.3)	321	(37.3)	127	(24.1)
Average weekly working hours (Mean, SD)	45.4	(9.7)	46.4	(9.7)	43.8	(9.6)
Employment status						
Practice owner (*n*, %)	1,106	(79.6)	679	(78.9)	427	(80.9)
Employed (*n*, %)	283	(20.4)	182	(21.1)	101	(19.1)
Type of practice						
Solo practice (*n*, %)	773	(55.9)	451	(52.8)	322	(61.0)
Other (*n*, %)	609	(44.1)	403	(47.2)	206	(39.0)
Self-perceived workload						
Low (*n*, %)	401	(29.0)	209	(24.4)	192	(36.6)
High (*n*, %)	982	(71.0)	649	(75.6)	333	(63.4)

Notes: Missing data: age (*n* = 12, 0.9%), average weekly working hours (*n* = 16, 1.2%), sex (*n* = 73, 5.3%), type of federal state (*n* = 50, 3.6%), employment status (*n* = 0, 0.0%), type of practice (*n* = 7, 0.5%), perceived workload (*n* = 6, 0.4%).

Female GPs (respondent = 67%) versus overall GPs (58%) and Gyns from rural states (respondent 46 % for Berlin, 29% for Brandenburg, 25% for Mecklenburg-Western Pomerania) versus overall Gyns (54% for Berlin, 27% for Brandenburg, 19% for Mecklenburg-Western Pomerania) were more likely to respond. Therefore, a weighting of data by sex and federal state was required, and performed, to ensure representativeness for the region.

### Collaboration amongst GPs and Gyns with regard to three key competencies for collaboration

For the 13 aspects of the 3 key competencies for collaboration, we found a similar perception in relation to ‘work effectively with physicians’, but deviations in ‘continuity of safe patient care’ and ‘promote understanding’. Some examples are listed below with the full list given in Table 2.

**Table 2.** Collaboration amongst GPs and Gyns based on aspects of key competencies

	All respondents (*n* = 1389)		General practitioners (*n* = 861)		Gynaecologists (*n* = 528)	
Aspects of collaboration	*n*	%	*n*	%	*n*	%
Work effectively with physicians						
Specialist available for consultation	745	(55.1)	468	(55.5)	277	(54.3)
Phone consultations with Gyn/GP	262	(19.2)	157	(18.6)	105	(20.2)
Advice to Gyn/GP	1,203	(88.0)	755	(89.3)	448	(85.8)
Advice from Gyn/GP	838	(63.0)	516	(62.5)	322	(63.8)
Continuity of safe patient care						
Agreement on indicators	67	(5.0)	42	(5.1)	25	(4.9)
Issues formal referral to Gyn for risk factors/formal referral from GP	876	(64.3)	643	(76.4)	233	(44.7)
Sends test results/working questions to Gyn/GP	667	(48.9)	485	(57.4)	182	(35.0)
Receives test results/working questions from Gyn/GP	450	(33.2)	304	(36.3)	146	(28.1)
Send doctor’s notes	245	(18.0)	46	(5.5)	199	(38.3)
Promote understanding						
No time for collaboration	505	(38.0)	307	(37.4)	198	(38.9)
Not aimed for by Gyn/GP	396	(31.6)	266	(34.7)	130	(26.6)
Satisfied with current collaboration	650	(51.9)	439	(57.2)	211	(43.5)
Change required	491	(38.8)	308	(39.8)	183	(37.1)

Notes: GP = General practitioner; Gyn = Gynaecologist.

#### Work effectively with physicians

Fifty-five per cent of respondents reported having a collaborating specialist of the other field for mutual consultation, whereas 19% of respondents reported consulting with each other on the phone.

#### Continuity of safe patient care

Only 5% of physicians reported having an established agreement on indicators for the mutual referral. In a country where there is no common patient electronic health record for multiple ambulatory treating physicians, doctor’s notes have particular importance in terms of multidisciplinary consultation to ensure care continuity. Amongst Gyns, 38% sent doctor’s notes to GPs, whereas only 6% of the GPs reported sending doctor’s notes to Gyns. For patients with risk factors, for example, high blood pressure and diabetes, 76% of the GPs referred the patient to a Gyn, whereas 45% of the Gyns reported receiving patients referred by the GPs.

#### Promote understanding

Satisfaction with current collaboration regarding women aged ≥ 50 years was reported by 57% of the GPs and by 44% of the Gyns, whereas 16% of the GPs and 12% of the Gyns were not satisfied with their current collaboration. Amongst all respondents, 38% reported the barrier of not having enough time to work together. A change in current collaboration practice was considered necessary by 40% of GPs and 37% of Gyns.

### Factors influencing collaboration amongst GPs and Gyns

Strong factors influencing collaboration are networking (member of a network versus not being member of a network) and the type of federal state (rural versus urban). In addition, the kind of specialist, the type of practice, and average weekly working hours are also of importance. Perceived workload and employment status showed an association for a few aspects (Table 3).

**Table 3.** Factors influencing the collaboration of GPs and Gyns (multiple logistic regressions)

Aspects of collaboration and factors of influencing	Unadjusted		Adjusted for age and sex	
	OR	95% CI	OR	95% CI
Work effectively with physicians^*^				
Specialist available for consultation				
Gyn (ref. GP)	0.76	(0.59, 0.99)	0.69	(0.53, 0.91)
Networking (ref. no networking)	8.16	(6.18, 10.78)	8.17	(6.11, 10.92)
Rural federal state (ref. urban)	2.16	(1.69, 2.77)	2.33	(1.80, 3.02)
Phone consultations with Gyn/GP				
Networking (ref. no networking)	3.50	(2.36, 5.18)	3.49	(2.32, 5.24)
Rural federal state (ref. urban)	2.23	(1.65, 3.01)	2.43	(1.77, 3.32)
Advice from Gyn/GP				
Networking (ref. no networking)	1.34	(1.06, 1.69)	1.43	(1.12, 1.83)
Continuity of safe patient care				
Agreement on indicators				
Networking (ref. no networking)	3.20	(1.57, 6.54)	2.76	(1.34, 5.68)
Other types of practice (ref. solo practice)	0.48	(0.26, 0.88)	0.50	(0.26, 0.95)
Average weekly working hours	1.04	(1.01, 1.06)	1.03	(1.00, 1.06)
Employed (ref. practice owner)	2.42	(1.16, 5.03)	2.74	(1.29, 5.81)
Issues formal referral to Gyn for risk factors/Formal referral from GP				
Gyn (ref. GP)	0.25	(0.20, 0.32)	0.24	(0.18, 0.31)
Networking (ref. no networking)	1.55	(1.19, 2.01)	1.53	(1.17, 2.00)
Rural federal state (ref. urban)	1.32	(1.04, 1.69)	1.40	(1.09, 1.80)
Average weekly working hours	1.02	(1.01, 1.03)	1.02	(1.01, 1.03)
Sends test results/working questions to Gyn/GP				
Gyn (ref. GP)	0.36	(0.28, 0.46)	0.33	(0.25, 0.42)
Networking (ref. no networking)	1.60	(1.25, 2.05)	1.53	(1.18, 1.98)
Rural federal state (ref. urban)	1.50	(1.19, 1.89)	1.61	(1.26, 2.06)
Other types of practice (ref. solo practice)	0.71	(0.56, 0.91)	0.71	(0.56, 0.92)
Average weekly working hours	1.02	(1.01, 1.03)	1.02	(1.01, 1.04)
Receives test results/working questions from Gyn/GP				
Gyn (ref. GP)	0.60	(0.46, 0.77)	0.62	(0.47, 0.81)
Networking (ref. no networking)	2.22	(1.68, 2.92)	2.16	(1.62, 2.87)
Rural federal state (ref. urban)	1.69	(1.32, 2.16)	1.80	(1.39, 2.32)
Other types of practice (ref. solo practice)	0.70	(0.55, 0.90)	0.72	(0.56, 0.94)
Send doctor’s notes				
Gyn (ref. GP)	11.06	(7.64, 16.03)	11.46	(7.78, 16.87)
Rural federal state (ref. urban)	2.41	(1.73, 3.35)	2.56	(1.82, 3.59)
Promote understanding				
No time for collaboration				
Networking (ref. no networking)	0.59	(0.47, 0.76)	0.60	(0.47, 0.77)
Rural federal state (ref. urban)	0.58	(0.46, 0.73)	0.54	(0.42, 0.68)
Perceived high workload (ref. low)	1.60	(1.23, 2.08)	1.65	(1.26, 2.17)
Not aimed for by Gyn/GP				
Networking (ref. no networking)	0.38	(0.29, 0.49)	0.37	(0.28, 0.48)
Rural federal state (ref. urban)	0.69	(0.52, 0.86)	0.64	(0.49, 0.82)
Satisfied with current collaboration				
Gyn (ref. GP)	0.55	(0.43, 0.70)	0.56	(0.43, 0.72)
Networking (ref. no networking)	2.98	(2.28, 3.89)	2.87	(2.17,3.79)
Rural federal state (ref. urban)	1.51	(1.18, 1.92)	1.61	(1.25, 2.06)
Other types of practice (ref. solo practice)	1.31	(1.02, 1.67)	1.41	(1.09, 1.82)
Change required				
Networking (ref. no networking)	0.57	(0.45, 0.74)	0.60	(0.46, 0.77)
Rural federal state (ref. urban)	0.52	(0.41, 0.66)	0.51	(0.40, 0.65)
Perceived high workload (ref. low)	1.40	(1.08, 1.82)	1.49	(1.13, 1.96)

Notes: OR = odds ratio; CI = confidence interval; GP = General practitioner; Gyn = Gynaecologist.

* For the model ‘Do you advise Gyn/GP consultation for your patients if necessary?’, no influencing factors could be found.

#### Work effectively with physicians

Effective collaboration amongst GPs and Gyns was associated with networking and the type of federal state demonstrated to be strong predictors. No influencing factors could be determined for the advice of the visit of the respective other specialists with an agreement rate of 88%. Physicians being members of a network reported significantly more often the presence of a colleague of the other specialisation for consultations [OR = 8.2 (6.1, 10.9), *P* < .001] and telephone calls [OR = 3.5 (2.3, 5.2), *P* < .001] than physicians not being members of any network. Furthermore, GPs and Gyns in a network received more advice from their counterparts [OR = 1.4 (1.1, 1.8), *P* = .004]. Physicians working in a rural federal state make phone calls about their patients more often than those working in an urban one [OR = 2.4 (1.8, 3.3), *P* < .001]. Type of practice, average weekly working hours, perceived workload, and employment status were not associated with ‘work effectively with physicians’ (Table 3).

#### Continuity of safe patient care

All examined variables except perceived workload showed an influence on the continuity of safe patient care. Stronger predictors were networking and the type of federal state. Physicians in a network reported having received test results or working questions more frequently than physicians without a network [OR = 2.2 (1.6, 2.9), *P* < .001]. Referrals for patients with risk factors are more common in rural federal states [OR = 1.4 (1.1, 1.8), *P* = .009] than in the urban one, but they are less frequent from Gyns [OR = 0.2 (0.2, 0.3), *P* < .001] than from GPs. Other types of practice are associated to a lesser degree with the variables of continuity when compared to solo practices, that is, continuity being examined by sending test results or working questions [OR = 0.7 (0.6, 0.9), *P* = .009]. A higher expression of examined variables is also accompanied by an indication of higher average weekly working hours, for example, agreement of indicators [OR = 1.0 (1.0, 1.1), *P* = .025, Table 3].

#### Promote understanding

Networking and type of federal state are predictors for barriers, as well as for satisfaction with current collaboration regarding women aged ≥ 50 years and the desire for change. The more objective criterion of average weekly working time and the employment status do not appear to be relevant, but the more subjective perceived workload is. Networking is associated with lower barriers, higher satisfaction, and minor change request than no networking, for example, no time for collaboration [OR = 0.6 (0.5, 0.8), *P* < .001]. The same applies to rural federal states in comparison to the urban one, for example, change required [OR = 0.5 (0.4, 0.7), *P* < .001]. Gyns are less satisfied with the current collaboration than GPs [OR = 0.6 (0.4, 0.7), *P* < .001]. Perceived high workload is associated with the barrier ‘no time for collaboration’ [OR = 1.7 (1.3, 2.2), *P* < .001] and with change request [OR = 1.5 (1.1, 2.0), *P* = .005, Table 3].

## Discussion

Collaboration to deliver healthcare services for women aged ≥ 50 years is more systematic amongst GPs and Gyns working in primary care providing outpatient health care in rural federal states of the North-Eastern region of Germany and between those who are member of a network. An increase in networking could lead to improved collaboration amongst GPs and Gyns with the key competencies ‘work effectively with physicians’, ‘continuity of safe patient care’, and ‘promote understanding’, and considering the importance of the continuity of care and collaboration, to ultimately help achieve improved outcomes for their patients.

### Work effectively with physicians

Strategies discussed with stakeholders to address shortages and the uneven distribution of the health workforce include the use of telemedicine, delegation, substitution, and interprofessional practice (Neumann *et al.*, 2014; Advisory Council on the Assessment of Developments in the Healthcare Sector, 2018). With reference to ‘work effectively with physicians’, previous studies found that specialists providing patient-centred care as members of interdisciplinary teams have led to an improvement in healthcare provision contributing to improved public health and towards a reduction in costs for healthcare services (Pollack *et al.*, 2012; Hussain *et al.*, 2015). Telemedicine is expected to play an important role as the digitalisation of healthcare advances, and also in the context of optimising workflows (Berger *et al.*, 2018; Albrecht *et al.*, 2018; Köhler *et al.*, 2019; Achenbach, 2019; Hagge *et al.*, 2020). Intensive collaboration between physicians is also assumed to be essential for the further introduction of telemedicine (Garattini *et al.*, 2020). In the field of women’s health, Dutch GPs agreed that collaboration between GPs and Gyns plays an important role in the early detection of endometriosis (van der Zanden *et al.*, 2020). In our study, both specialist groups advise consultations with the other speciality as a contributing factor to establishing a solid foundation for effective collaboration and the continuity of safe patient care.

Since 2005, physicians in Germany can no longer reimburse a telephone consultation taking place between colleagues (National Association of Statutory Health Insurance Physicians, 2004; 2005). In our study, GPs and Gyns in rural areas reported consulting a colleague via telephone more often than their colleagues in urban areas. This shows the need for more interaction and communication with colleagues. Telephone is one of the preferred instruments for interaction in these areas. Consideration should be given to whether a reintroduction of the billing facilities for telephone consultations could lead to an improvement in collaboration amongst GPs and Gyns.

### Continuity of safe patient care

The CanMEDS framework has recently been incorporated into the curriculum of the general practice residency programme in Germany (Steinhäuser *et al.*, 2013; Flum *et al.*, 2015). However, considering the average age of German GPs, the scope of their speciality training and the time they received, as well as the general continuous medical education and accreditation regulations – which do require a certain number of hours, but without stipulating specific contents – it can be assumed that the level of awareness for the competencies of collaboration is low.

The importance of collaboration in the ‘continuity of safe patient care’ was demonstrated by the example of a greater frequency of HIV testing, HIV primary care, and psychoeducation (Pinto *et al.*, 2018). In Italy, agreed pathways for referral to specialists for headaches are considered a quality feature of treatment for both normal and urgent cases (Pellesi *et al.*, 2017). The present study demonstrated that in Germany, most of the GPs regularly refer patients with gynaecological risk factors to Gyns, but there is no established agreement on specific indicators for when a referral or a joint consultation is required. Clinical guidelines have been developed by the German Society for Gynaecology and Obstetrics with the participation of the German Society of General Medicine and Family Medicine including for the management of breast cancer and menopause (German College of General Practitioners and Family Physicians, 2017a; 2017b). So far, in Germany, there are no clinical pathways concerning the management of women’s health for women aged ≥ 50 years offering guidance on establishing and navigating collaboration amongst GPs and Gyns.

We found that doctor’s notes were rarely exchanged between treating physicians. In the case of GPs, this could also be confirmed via billing data (Stillfried *et al.*, 2017). We did not explore the timeliness of sending or receiving reports, but previous studies have shown even in the case notes/results were sent, they were often sent late (Lang *et al.*, 2018). There is a current debate on the introduction of digital health records and the patients’ rights (Advisory Council on the Assessment of Developments in the Healthcare Sector, 2018). Available electronic health records could make all medical reports and results accessible for co-treating practitioners (Lang *et al.*, 2018). Further studies are needed to explore the effect of a stronger collaboration on the quality of patient care and management in Germany, but given GP shortages, commonalities in reporting challenges, and the continuous pressure on the system, such studies are needed across Europe.

### Promote understanding

Having no time for collaboration was the barrier reported mostly amongst physicians working in the urban federal state, who additionally did not consider there was interest for collaboration from their counterpart. Both barriers were more frequently reported amongst physicians not being member of a network.

Overall, we found a high proportion of physicians who are dissatisfied with the current collaboration culture and/or considered a change indicating the need for action. Physicians satisfied with the collaboration are more often GPs from rural areas who are members of a network.

Further studies should explore these relationships and their connection with participation in networks, however, it is clear that investing in networking is key. Networking is seen as part of the professional identity of physicians in outpatient care, which is both important and feasible (Bertin & Pantalone, 2019). Supporting networking and personal relationship could be one aspect of how to increase collaboration amongst physicians (Berendsen *et al.*, 2007). This is particularly important for the provision of care at the primary care level for women aged ≥ 50 years. The results of this study show that it is also necessary to further examine influencing parameters, including though implementation research to allow assessment of contextual factors, as, for example, the feasibility of establishing innovative ways of network formation and participation, digital tool utilisation, and exploring the role improved workflows across levels of care can play to improve outcomes.

Furthermore, special consideration should be given to women aged ≥ 50 years because of the proportion of the total population they currently represent, and the fact their number is expected to rise significantly over the next 10 years. An increasing number of women aged ≥ 50 years is predicted not only for rural areas in Germany, but on a worldwide level, including across developed countries (United Nations *et al.*, 2019; Federal Statistical Office of Germany, 2019).

### Strengths and limitations

This quantitative study explores the self-reported perspective of the current state of collaboration, including barriers and change requests amongst GPs and Gyns in Germany’s primary care in relation to women aged ≥ 50 years for the first time. It reflects only the perspective of the participating physicians, whereas the perspective of the patients should also be explored systematically in the future.

A strength of the survey was the high response rate of 51% amongst Gyns, which was partly achieved thanks to the support of the Professional Association of Gynaecologists.

In terms of limitations, we cannot rule out response bias or answers of social desirability. Coverage and selection bias were taken into account when designing the survey and selecting the sample of GPs to the extent possible, that is, with piloting the questionnaire and using an updated framing sample list.

In order to reduce response bias and to ensure the transferability of the results across the North-Eastern region, outcomes were weighted according to federal state and sex. The transferability of the results beyond the investigated North-Eastern region is only possible to a limited extent. The results of the study cannot be interpreted causally or as associations.

## Conclusion

Multiprofessional collaboration amongst primary care providers should include different medical specialties, as well as other healthcare professionals. Strengthening this collaboration and its key competencies through networking can contribute towards the continuity of safe patient care, promote understanding, and ultimately, ensure a patient-centred and lifecycle-appropriate healthcare service provision to women ≥ 50 years. Furthermore, tailored integration of collaborative primary care services can be a core component of mitigation strategies to counter compromised access and to bridge inequalities between urban and rural settings, ultimately, contributing to patient safety and quality improvement. It can also inform practice-based research and cross-regional collaboration so as to better serve the needs of patients and of practitioners alike.
